# Closing the knowledge gap on the composition of the asbestos bodies

**DOI:** 10.1007/s10653-023-01557-0

**Published:** 2023-04-14

**Authors:** F. Bardelli, C. Giacobbe, P. Ballirano, V. Borelli, F. Di Benedetto, G. Montegrossi, D. Bellis, A. Pacella

**Affiliations:** 1 National Research Council, Institute of Nanotechnology (CNR-Nanotec), Rome, Italy; 2 Centre for the Study of Asbestos and Other Toxic Particulate, University of Torino, Turin, Italy; 3 Xenocs SAS, Grenoble, France; 4 European Synchrotron Radiation Facility, Grenoble, France; 5 Department of Earth Sciences, La Sapienza University, Rome, Italy; 6 Department of Physiology and Pathology, University of Trieste, Trieste, Italy; 7 Department of Earth Sciences, University of Ferrara, Ferrara, Italy; 8 National Research Council, Institute of Geoscience and Earth Resources (CNR-IGG), Florence, Italy

**Keywords:** Asbestos bodies, Iron, Ferrihydrite, Goethite, Mesothelioma, Toxicology, Synchrotron radiation

## Abstract

Asbestos bodies (AB) form in the lungs as a result of a biomineralization process initiated by the alveolar macrophages in the attempt to remove asbestos. During this process, organic and inorganic material deposit on the foreign fibers forming a Fe-rich coating. The AB start to form in months, thus quickly becoming the actual interface between asbestos and the lung tissue. Therefore, revealing their composition, and, in particular, the chemical form of Fe, which is the major component of the AB, is essential to assess their possible role in the pathogenesis of asbestos-related diseases. In this work we report the result of the first x-ray diffraction measurements performed on single AB embedded in the lung tissue samples of former asbestos plant workers. The combination with x-ray absorption spectroscopy data allowed to unambiguously reveal that Fe is present in the AB in the form of two Fe-oxy(hydroxides): ferrihydrite and goethite. The presence of goethite, which can be explained in terms of the transformation of ferrihydrite (a metastable phase) due to the acidic conditions induced by the alveolar macrophages in their attempt to phagocytose the fibers, has toxicological implications that are discussed in the paper.

**Supplementary Information:**

The online version contains supplementary material available at 10.1007/s10653-023-01557-0.

## Introduction

Asbestos is long recognized as a group 1 human carcinogen (Overall Evaluations of Carcinogenicity, 1987), and, besides other serious pulmonary diseases, such as asbestosis, it induces pleural mesothelioma, a highly lethal cancer of the lung lining, with a five-year survival rate of only five percent (Shavelle et al., 2017). The first scientific evidences linking asbestos to mesothelioma date back to the sixties, and were first based on epidemiological studies and then on in vitro and in vivo experiments on cell cultures and animal models (Ghio et al., 2008; Governa et al., 1999; Wagner et al., 1974). However, despite beginning from the eighties numerous countries started to ban asbestos extraction, production, and commercialization, asbestos continue to pose a major health threat. Over 7000 deaths per year on average are estimated for the period 1995–2029 (Peto et al., 1999) only in Western Europe. The main reason why asbestos is still a current danger worldwide is the long latency time of mesothelioma, which falls in the range between 20 and 40 years, or more, from the first exposure. Other reasons can be attributed to the weathering of the enormous amount of existing asbestos-containing materials (ACM: e.g., roof tiles, pipes, water tanks, and others), to the fact that asbestos is not banned in several countries (e.g., China, Brazil, Canada, Russia), and to naturally occurring asbestos (NOA) (Bloise et al., 2016).

**Fig. 1:**
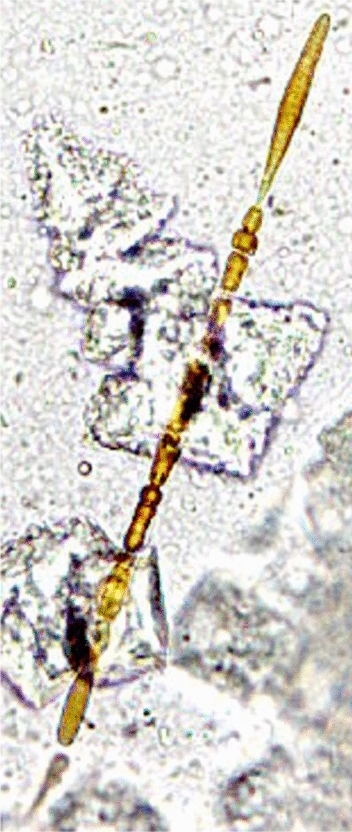
Optical microscope image of a representative AB recovered by chemical digestion from the lung tissue samples studied in this work (original magnification ×400)

In the lungs, a biomineralization process is initiated by the alveolar macrophages during their attempt to remove the foreign fibers by phagocytosis (Bardelli et al., 2017; Hamilton et al., 1996). Following repeated phagocytosis attempts, minerals and proteins deposit in layers on the surface of the asbestos fibers (Bursi Gandolfi et al., 2016), resulting in the formation of peculiar structures indicated as asbestos bodies (AB) (Fig. 1). Due to their small dimensions, (diameter of 1–6 μm and a length determined by the underlying mineral fiber, usually 10–100 μm), and to the complexity of obtaining sufficient quantities, it is extremely difficult to study the composition of the AB by conventional analytical techniques, such as, for example, Inductively Coupled Plasma—Mass Spectroscopy (ICP-MS, (Nakamura et al., 2009)), without the risk of altering them. For this reason, their nature and composition remained unclear, preventing the determination of their role in the pathogenesis of asbestos-related diseases.

They have long been considered as inert bodies, isolating the biological tissue from asbestos (Governa et al., 1999; McLemore et al., 1980). However, they have been shown to induce DNA damages (Lund et al., 1994), production of reactive oxygen species (ROS) (Ghio et al., 2008), and to be catalytically active (Fubini & Mollo, 1995), suggesting that they may even enhance the cytotoxic properties of asbestos.

Due to their microscopic dimensions, the AB were usually studied by scanning or transmission electron microscopies (SEM or TEM). Pioneering TEM studies revealed that they are mainly composed of Fe (Gloyne, 1931), and that the size of the granular material composing the coating is compatible with that of the ferritin protein (Davis, 1964; Suzuki & Churg, 1969). Selected area electron diffraction (SAED) measurements performed by Pooley on single AB, revealed d-spacings compatible with the presence of ferritin or hemosiderin (Pooley, 1972).

However, electron microscopies usually require the removal of the biological tissue for good imaging and microprobe analyses (e.g., energy-dispersive spectroscopy (EDS)). This can be achieved by different methods, the most common being chemical digestion in KOH or NaClO, or ashing. However, one major issue that emerged already in the first studies is that these methods may alter the chemical composition and structure of the AB. In fact, already in 1972, Pooley reported that the structures of the AB recovered by ashing or by alkali digestion were different (Pooley, 1972).

In 2007, Borelli et al. have first been successful in studying the AB in a more biologically representative form by recovering the AB with a magnetic separation method (Borelli et al., 2007). Contrary to the chemical digestion or ashing, this method was claimed to prevent any alteration of the AB. With this innovative approach they were able to obtain enough material to analyze the proteinaceous composition of the coating using Peptide Mass Fingerprint (PMF) analysis of the electrophoretic bands, finding the signature of ferritin, albumin, and possibly hemosiderin. In addition, they found that the AB extracted by magnetic separation and those extracted by chemical digestion have different behavior toward inflammatory cells. In particular, magnetically recovered AB were able to induce a higher production of free radicals, while, unexpectedly, no DNA damage was observed, contrary to the AB recovered by chemical digestion. These results confirmed that the methods used to recover the AB might alter their cytotoxic properties, and, therefore, conclusions drawn from studies performed on AB recovered with invasive sample preparation approaches must be considered carefully.

A full understanding of the role of Fe in the pathogenesis of mesothelioma can only be achieved with the knowledge of the chemical form that Fe assumes in the AB, as, in the long term, these become the actual interface between the asbestos and the biological tissue. However, until now, studies suggesting that the AB are mainly made of ferritin were indirect or not conclusive. In fact, one of the main evidences in favor of ferritin derived from the observation that the AB can also form on Fe-poor asbestos, as chrysotile, leading to the conclusion that the source of the Fe contributing to the AB growth must be endogenous (i.e., from the host organism) (Giacobbe et al., 2021; Suzuki & Churg, 1969). Therefore, the assumptions that the Fe-rich protein ferritin was the main source of Fe seemed reasonable, as the alveolar macrophages host ferritin and are believed to play a major role in the formation of the AB. Electron diffraction studies, which suggested the presence of ferritin (Di Giuseppe et al., 2019; Pooley, 1972), cannot be considered conclusive as well, because they were performed on AB recovered by invasive method (i.e., chemical treatments or plasma ashing), and because of the possible beam damage induced by the electron beam, which may have altered the structure of the samples. On the other hand, the determination of the protein contribution in the AB revealed by Borelli et al. (Borelli et al., 2007) on AB obtained with less-invasive methods, cannot unambiguously reveal the Fe speciation. In fact, despite it is known that Fe is deposited inside the ferritin protein in the form of ferrihydrite (Harrison et al., 1967), this phase is metastable, and, as it will be discussed in the following, it may evolve to other Fe species.

Some authors exploited variable pressure SEM with annexed Energy-Dispersive Spectroscopy (EDS) and/or micro-Raman spectroscopy to study AB in thin sections of lung tissues (Croce et al., 2013; Rinaudo et al., 2010). However, even if these approaches do not require invasive sample preparation, the authors claimed that the laser beam of the spectrometer may have induced photo-crystallization of hematite in the Fe-rich material forming the AB.

During the next decade, advanced x-ray imaging and spectroscopic techniques, based on synchrotron radiation, allowed to study single AB embedded in the original lung tissue, limiting the sample preparation to formalin-fixing and paraffin-embedding the asbestos-containing tissues. This approach, along with the negligible beam damage of x-rays compared to electron beams, minimized the possibility of altering the AB. In particular, high resolution scanning x-ray fluorescence mapping revealed a different elemental composition of AB extracted by chemical digestion and those studied in the biological tissue, and x-ray absorption spectroscopy (XAS) suggested that Fe is mainly present in the form of ferrihydrite (Bardelli et al., 2017), the mineral core of ferritin (Harrison & Arosio, 1996). However, another study reported XAS measurements that suggested the presence hematite and metallic Fe in the AB, along with ferritin (Pascolo et al., 2013).

The results of the above cited works show that, despite much effort and different techniques were applied to answer the problem of the AB composition, no consensus has been reached yet.

In this work, we combined the results of x-ray diffraction (XRD) and XAS measurements to study single AB embedded in the original lung tissue, in the attempt to obtain a conclusive determination of the Fe-form in the AB.

## Materials and methods

### Lung tissue samples

Non-neoplastic lung tissues of two former workers of an asbestos-cement plant that operated between 1946 and 1982 in North-Western Italy (Piedmont region), were obtained post-mortem from the lower lobes of the lungs. The workers were subjected to prolonged exposure to different type of asbestos, mainly chrysotile, but also crocidolite and amosite, and had asbestosis, pleural-plaques, and mesothelioma or lung cancer (Table 1).

**Table 1:** Lung tissue samples studied in this work

Case	Age	Sex	Exposure duration	Diagnosis
A	81	M	27 years	^a^AS (grade 3), ^b^PP, and ^c^MM
B	80	F	> 10 years	AS (grade 4), PP, and ^d^LC

^a^AS: Asbestosis (grading established according to Craighead et al. (1982)); ^b^PP: pleural plaques; ^c^MM: pleural mesothelioma; ^d^LC: lung cancer

The estimation of the AB count per gram of dry tissue and per unit volume (cm^3^), and of their size distribution in the same tissue samples can be found elsewhere (Bardelli et al., 2021a), and a study of their elemental composition and morphology is reported in (Bardelli et al., 2021b).

The lung tissue samples were preserved in buffered formalin (10%) until measurements. Tissue fragments of size of about 100 µm in diameter and containing a single AB were cut from 10 µm-thick tissue sections using a laser micro-dissector. The fragments were then deposited on Ultralene^®^ films for XAS measurements, or glued on MiTeGen Microloops for x-ray diffraction measurements (XRD) ones (Figure S1, Supporting Information).

### X-ray absorption spectroscopy

XAS is informative of the local structure and of the oxidation state and site geometry of a selected atomic species (Lee et al., 1981) (i.e., Fe in this work). The Fe K-edge X-ray Absorption Near-Edge Structure (XANES) spectra of eight AB embedded in the lung tissue were acquired in transmission and fluorescence modes at the ID21 beamline at the European synchrotron (ESRF) in Grenoble. Two to four regions of interest (ROI) of the AB were measured with an X-ray beam of size 0.5 µm × 0.5 µm. The Fe references measured included horse spleen ferritin, magnetite, hematite, ferritin, hemin, haematin, and Fe(II)-l-ascorbate (Sigma-Aldrich).

The spectra of goethite, lepidocrocite, magnetite, hematite, ferritin (Merck), and ferrihydrite were acquired in transmission mode at the B18 beamline at the Diamond Light Source, using a beam spot size of 2.0 µm × 2.0 µm. The ferrihydrite reference was synthesized by precipitation from a 0.1 M FeCl_3_ solution in 0.01 HCl through pH increase by adding NaOH 1 M drop to drop (Jia et al., 2007). The precipitate was filtered, washed, and let dry at room temperature.

The XANES spectrum of a crocidolite standard from the Union for International Cancer Control (UICC) was also measured.

The same monochromator crystals (Si 111) and energy steps (~ 0.3 eV) were used at all ID21 and B18 beamlines, ensuring that the spectra have similar energy resolution. The energy scale of the all reference spectra was calibrated using the spectrum of a Fe foil mounted behind the samples, which was acquired simultaneously at each energy scan. For energy calibration of the AB spectra, a spectrum of an Fe foil was measured before and after each energy scan. The spectra of all Fe reference are reported in Figure S2 in the supporting information). All spectra were background subtracted and normalized using the Demeter software package (Ravel & Newville, 2005). The number of samples and XAS measurements are summarized in Table S2 in the Supporting Information.

### X-ray diffraction

X-ray diffraction measurements can reveal the crystallographic structure(s), and thus the mineral phase(s) present in the sample. The measurements were performed at the ID11 beamline at the ESRF on different ROI of three AB. The sample was able to rotate on a high-precision air-bearing axis and the sample alignment on the rotation axis was achieved using a hexapod stage mounted on a piezoelectric stage with a minimum incremental movement of 0.1 µm in the three orthogonal directions. Diffraction data were acquired using an X-ray beam of size 0.15 µm × 0.15 µm and a wavelength of 0.28528 Å, which was obtained via a bent Si (111) Laue–Laue double-crystal monochromator. Images with 2048 × 2048 pixels were acquired using an Eiger2 CdTe 4 M detector (Dectris) with 75 µm × 75 µm pixel size. The sample-to-detector distance (124.8 mm) and tilts were calibrated using a CeO_2_ powder standard. Rotation scans of the AB were recorded over an angular range of ± 10° about the vertical axis in steps of 0.25° and integrating for 20 s. The resulting patterns where then averaged to increase the statistics of the data. As the goethite measured at the synchrotron facility resulted not pure, another goethite reference (Merck) was measured in transmission mode on a capillary with a laboratory diffractometer (Bruker) at the Cu K*α* wavelength (1.5406 Å), along with a crocidolite reference from the UICC. The number of samples and XRD measurements are summarized in Table S2 in the Supporting Information.

### Thermodynamic calculations

Pourbaix diagrams (Eh vs. pH) were calculated according to the point-by-point mass balance method (Huang, 2016) to study the relationships between the main phases of the Fe–O–H system. This approach allows to identify the predominant species (i.e., the species with the highest Fe content) at each Eh and pH value, temperature, and pressure. Calculations were performed under the PHREEQC formalism (Parkhurst & Appelo, 1999), and the absolute Fe content (in moles) is considered for both aqueous and solid species. Aqueous phase equilibria were taken into account using a reference database (Blanc et al., 2012), and the solid phases were included through dissolution/precipitation reactions and reported as the logarithm of equilibrium constant (log K) at 37 °C and 1.0132 bar. Equilibrium calculations were carried out over an array of Eh–pH points (500 × 500), in the pH range 3–10, and in the entire stability field of water (Eh range: − 0.74 to 1.10 V). The initial Fe concentration, temperature, and pH were set to those typical of a healthy adult male (25 μm/L, 37 °C, and 7.4, respectively), while the O_2_ fugacity is controlled by the Eh value.

The hypersurfaces of the Saturation Index versus Eh and pH values for the solid species considered in the model (i.e., hematite, goethite, magnetite, and ferrihydrite) were calculated by: (a) allowing the solid species to precipitate according to their solubility, and (b) preventing all solid phases, but ferrihydrite, to precipitate. The second approach can reveal if the residual Fe content in solution (after the complete ferrihydrite precipitation), is still oversaturated, so that goethite, magnetite, or hematite are thermodynamically able to precipitate. The hypersurfaces are shown in 3D plots. The rendering of the 2D plots (predominance Pourbaix diagrams) and 3D plots (SI diagrams) was carried out with the OriginPro 2021b software.

## Results

Compared to previous studies, the use of X-ray techniques had three main advantages: (1) it allowed to measure the AB in the original lung tissue, (2) the x-rays interact much less with the samples with respect to the electron beam, reducing the risk of altering the AB (beam damage). This applies particularly for the energy used to perform XRD measurements (43.457 keV), which is much higher than the Fe absorption edge (7.112 keV), (3) a full diffraction pattern can be obtained, which removes the uncertainties arising from univocally attributing the few diffraction rings that can be obtained from selected area electron diffraction (SAED) to single phase(s).

### X-ray diffraction

X-ray diffraction on single AB has never been attempted before because of the low scattering power of single AB. The key for successful measurements was to work with a very intense and focused x-ray beam (0.15 μm × 0.15 μm), which allowed detecting the scattering from single AB. In addition, since the size of the x-ray beam was ten times smaller than the typical diameter of the AB, and comparable with the diameter of a single asbestos fiber (0.15 – 0.25 μm), it was possible to measure separately the diffraction signals from the inner fiber and from its ferruginous coating.

**Fig. 2:**
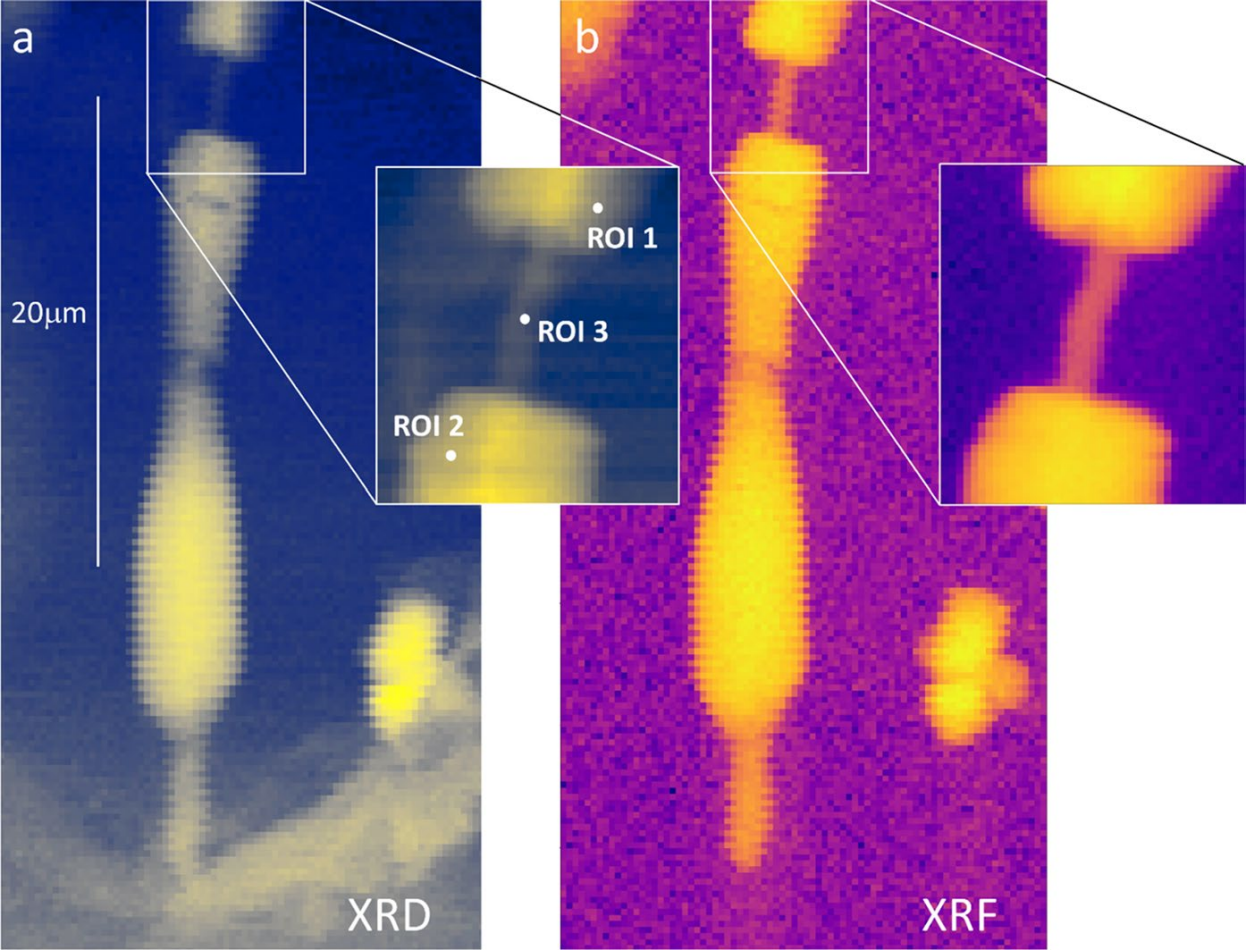
**a** Map of the total XRD signal of a representative AB embedded in the lung tissue. A diffraction image is associated with each pixel. The labelled white points correspond to point XRD acquisitions acquired by rotating the sample of a certain angle (see "X-ray diffraction" section). **b** XRF map acquired simultaneously to the XRD map and showing the distribution of Fe in the AB. The insets show higher resolution maps (pixel size 0.15 μm × 0.15 μm) of a smaller area. The incident x-ray beam energy was 43.457 keV

For each sample (*n* = 3), simultaneous X-ray fluorescence (XRF) and XRD mapping was performed to image the samples and allow choosing different ROIs for point XRD acquisitions. Representative XRF and XRD maps, showing, respectively, the total diffraction signal and the Fe distribution in an AB, are reported in Fig. 2. The AB, with the typical ferruginous coating is clearly visible in both maps, and an area containing the lung tissue is visible at the bottom of the XRD map. In the upper part of the maps (see inset of Fig. 2), it can be seen a region where the coating is missing, revealing an inner, Fe-bearing, mineral fiber.

**Fig. 3:**
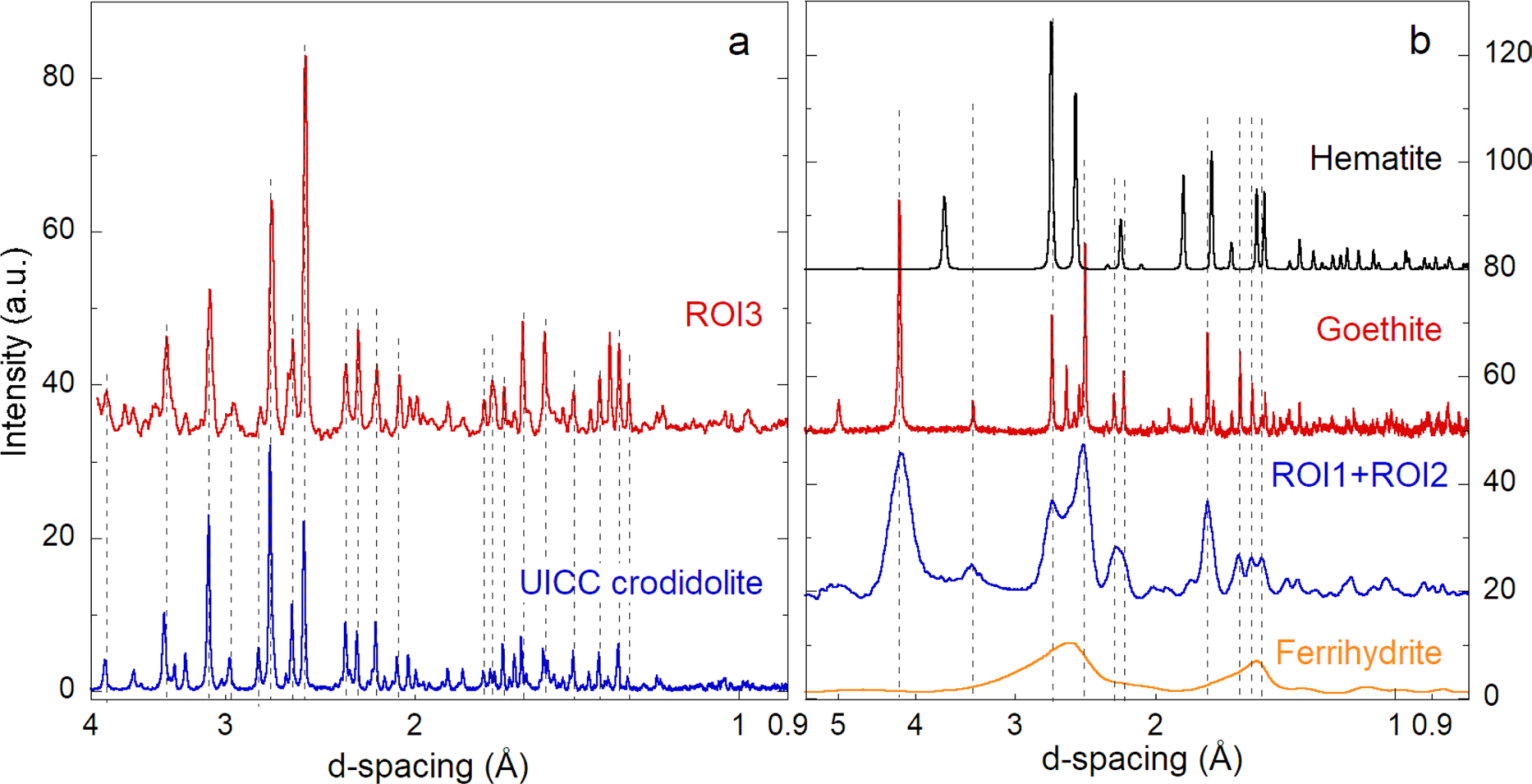
XRD patterns acquired on the ROIs indicated in Fig. 2 compared with reference patterns. **a** The XRD pattern acquired on an uncoated part of an AB (ROI3) matches that of the UICC crocidolite standard. **b** The average XRD pattern acquired at ROI1 and ROI2 (ROI1 + ROI2) matches the pattern of goethite. The synthetic ferrihydrite reference is closer to 6-line ferrihydrite than to the 2-line one (see text and Figure S5 in the Supporting Information). The dashed vertical lines highlight the main peak correspondences between data and references. The ROIs patterns and those of the hematite and ferrihydrite references were acquired at a wavelength of 0.2853 Å with an x-ray beam spot size of 0.15 μm × 0.15 μm. The reference patterns of goethite and crocidolite were acquired in transmission mode on a capillary with a laboratory diffractometer at the Cu K-α wavelength. The patterns have been background subtracted (using the pattern of an empty sample holder and a polynomial function to fit the residual background), rescaled, and vertically shifted for easy of view and comparison. The x-axes (d-spacing) are in logarithmic scale

The diffraction pattern resulting from the inner fiber (ROI3, Fig. 2a) is reported in Fig. 3a, and matches well with the reference pattern of crocidolite from the Union of International Cancer Center (UICC). This results confirms the high biopersistency of amphibole asbestos, which bulk structure remains unaltered even after decades in the biological tissues, as reported in recent studies (Di Giuseppe et al., 2019; Giacobbe et al., 2021; Gualtieri et al., 2017; Pollastri et al., 2016).

In Fig. 3b, the sum of the XRD patterns acquired on the Fe-coating (ROI1 and ROI2, Fig. 2a) is shown. As can be seen from the figure, the resulting pattern matches well with the reference pattern of goethite. The coherency domain of goethite, calculated with the Scherrer equation from the 110 reflection, is of ca. 8 nm, reflecting in the much larger width of the XRD peaks of the sample with respect to the goethite reference. The XRF/XRD maps of the other two samples are reported in Figures S3 and S4, along with the diffraction patterns associated to the different ROIs.

### X-ray absorption spectroscopy

**Fig. 4:**
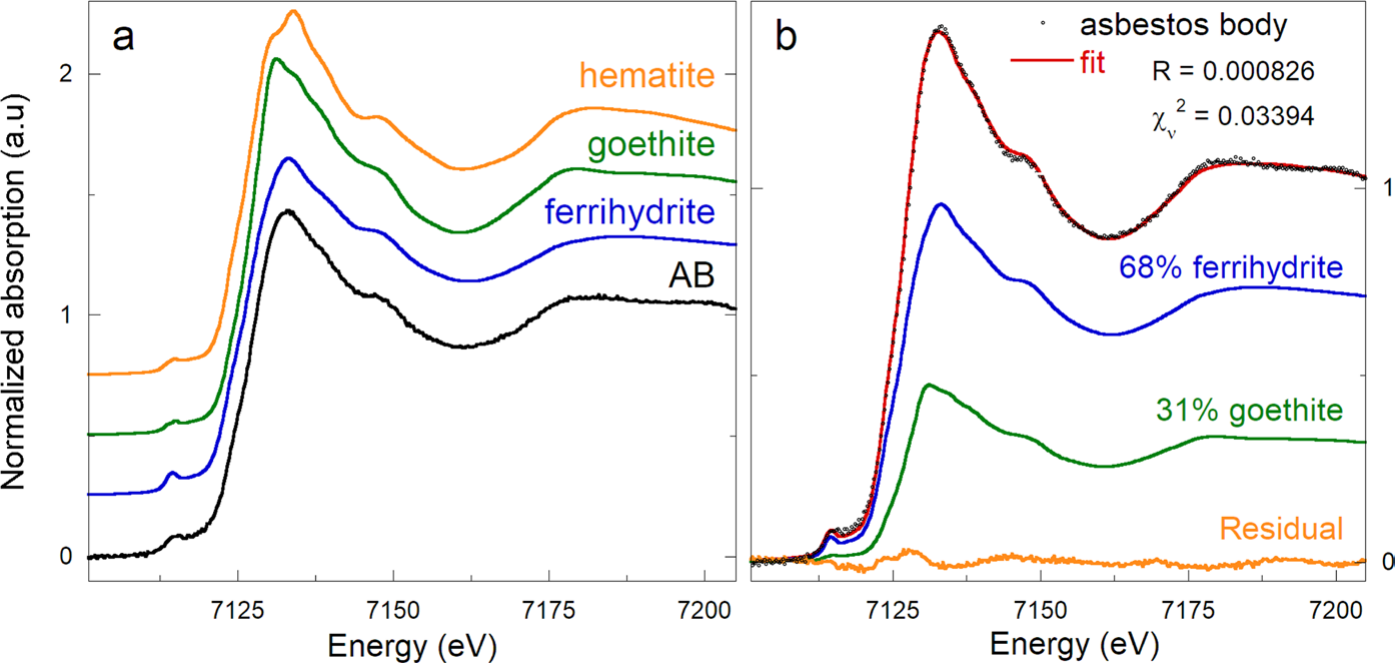
**a** XANES spectra of an AB and of the hematite, goethite, and ferrihydrite references. **b** Linear Combination Fit (solid red curve) of a spectrum of an AB (black points) performed using the ferrihydrite and goethite reference spectra (solid blue and green curves, respectively). Best fit is achieved by using, on average, ~ 68% ferrihydrite, and ~ 31% goethite. The estimated absolute error on the Fe-species is about 5%

As previous XAS studies did not reveal goethite, a new XAS experiment was performed, this time including goethite among the Fe references, along with hematite, lepidocrocite, magnetite, ferrihydrite, and ferritin. The results of this new experiment are shown in Fig. 4a, where the spectra of hematite, goethite, and ferrihydrite are compared with the spectrum of an AB resulting from the average of the spectra acquired at four ROIs. The ferritin and ferrihydrite spectra are very similar to that of the AB, while goethite and hematite have quite different spectral features. This is expected, because ferrihydrite is a poorly crystalline material, reflecting in a less structured spectrum than those of hematite or goethite. To check if it was possible to achieve a better match with the spectrum of the AB, Linear Combination Fitting (LCF) was attempted by using the Fe-oxides, hydroxides, and oxyhydroxides references to reproduce the spectrum of the AB. This approach can reveal the speciation of a selected element, provided that the set of reference spectra is representative of the studied system (Isaure et al., 2002).

Linear combination fits were attempted by considering all possible combinations of the set of Fe-reference spectra, and by keeping the relative energy scales of the samples spectra and references fixed. An *n* + 1 component combination was considered better than an *n* component one if the match with the sample spectrum improved significantly (i.e., if the statistical indicators *R* and reduced *χ*^2^ decreased more than the error associated with the LCF, which was estimated to be about 5% for 2-component LCF). The best fit was obtained with a 2-component linear combination using about 68 ± 8% ferrihydrite and about 31 ± 7% goethite, where the numbers after the values represent the standard deviation on four ROI of a representative AB (see Figure S6 and Table S1 in the Supporting Information). If hematite is included as a third component, its contribution to the LCF accounted for about 2% (Figure S7). However, considering that the fits do not improve significantly by including hematite, and that the amount of hematite falls below 5%, the possible presence of hematite cannot be confirmed by XAS data. However, a fraction of ~ 2% of hematite should have been observed in XRD data as well, which was not the case; therefore its contribution to the LCF is likely an artifact due to the addition of another free parameter in the fitting procedure.

With the exception of ferritin, which spectrum is almost identical to that of ferrihydrite (as expected, being ferrihydrite the mineral core of ferritin), all other Fe references did not contributed to the LCF, or, when used in place of ferrihydrite and/or goethite, the match with the experimental data was not good.

The results of these new XAS measurements suggest that previous XAS studies (Bardelli et al., 2017; Pascolo et al., 2013) failed to detect goethite in the AB because it was not included in the set of reference compounds.

## Discussion

The result of XRD measurements, which revealed the presence of goethite, was somehow unexpected, as previous studies converged on the ferrihydrite hosted by ferritin as the main component of the AB. However, the presence of goethite is compatible with the metastable nature of ferrihydrite, which is known to be a precursor of goethite and hematite (Cudennec & Lecerf, 2006; Das et al., 2011; Schwertmann et al., 2004). Furthermore, XRD measurements are only apparently in contrast with XAS ones, which detected ferrihydrite in addition to goethite, with the first being significantly more abundant than the other (roughly in the ratio 70:30). In fact, ferrihydrite can manifest with different levels of crystallinity, corresponding to the so-called 2- to 6-line ferrihydrite, which are the end members of a series of nano-crystalline phases with increasing size of the coherent domains. In particular, the less crystalline form, 2-line ferrihydrite, reflects in an XRD pattern where only two broad bands centered at 2.5–2.7 Å and at 1.5–1.7 Å are visible (Das et al., 2011). Therefore, if only poorly crystalline 2-line ferrihydrite were present in the AB, along with crystalline goethite, its characteristic weak peaks would be easily hindered by the two superimposed clusters of strong reflections of goethite (Figs. 3b and S5). On the other hand, XAS is not affected by the level of crystallinity of the samples, and is able to reveal the local structure also in disordered, nano-crystalline, or amorphous systems. This explains why ferrihydrite was detected by XAS and not by XRD, and highlights the complementarity of the two techniques in determining the actual speciation of Fe in the AB.

To check if the preparation of the lung tissue samples could have itself favored the transformation of ferrihydrite into goethite, synthetic ferrihydrite was prepared as described in the methods section and subjected to the same protocol used for fixation of the biological samples. Briefly, ferrihydrite powder was kept in buffered formalin solution (10%) for 72 h or 168 h, and then washed and filtered on porous membranes (0.45 μm pore size) and poured in ethanol solutions (70%, 95%, and 100%), and then in xylene. The samples were finally heated at 60 °C for 12 h (full details of the treatment can be found in the caption of Figure S8 in the Supporting Information). The powder was then recovered and XRD measurement performed in transmission mode on a capillary at the Cu Kα wavelength. The resulting patterns did not reveal the presence of goethite, confirming that the sample preparation did not affected the Fe-form in the AB (Figure S8).

The presence of ferrihydrite, and, to a lower extent, of goethite, as the major phases, and the absence of hematite must be contextualized and explained in terms of what is known on the stability of ferrihydrite, and on its transformation mechanisms to more crystalline Fe oxides, as goethite or hematite.

The hydrolysis of Fe^2+^ and Fe^3+^ ions in solution leads to the formation of Fe hydroxides, oxy-hydroxides, or oxides, depending on reaction conditions such as pH, temperature, concentration of Fe^2+^ and Fe^3+^, and concentration of other ions in solution (Cudennec & Lecerf, 2006). Although ferrihydrite is thermodynamically unstable, it is kinetically favored in presence of high concentration of Fe^3+^, such as during the rapid oxidation of dissolved Fe^2+^, as is the case of ferrihydrite nucleation inside the ferritin protein (Chasteen & Harrison, 1999). On the contrary, crystalline minerals with low solubility, such as goethite or hematite, can form ordered lattices only if their building blocks are supplied at a low rate (Carlson & Schwertmann, 1981). In Fe-rich and oxidative conditions, hydrolysis usually first leads to precipitation of ferrihydrite (5Fe_2_O_3_⋅9H_2_O), which can transform to goethite (*α*-FeO(OH)) or hematite (*α*-Fe_2_O_3_) following different transformation processes, in competition one with the other. Goethite is believed to form from ferrihydrite through a dissolution–crystallization process, while hematite by solid state transformation of ferrihydrite (Das et al., 2011). In particular, higher temperatures always favor the formation of the more crystalline oxide, hematite (Cudennec & Lecerf, 2006). Goethite formation is favored in the pH ranges between 4 and 5, where ferrihydrite reaches its maximum solubility, and between 10 and 14. Hematite, on the other hand, tends to form around near-neutral pH (6–9), in correspondence with the minimum solubility of ferrihydrite (Schwertmann et al., 2004).

In this work, we observed the presence of goethite, in addition to ferrihydrite, suggesting that the AB were exposed to pH conditions far from the near-neutral ones.

A possible scenario is that goethite forms during the macrophages (frustrated) attempt to ingest and eliminate asbestos. During this process, the macrophages secrete extracellular vesicles containing Fe-loaded ferritin that adsorbs on the fibers (Ito et al., 2021; Koerten et al., 1990b; Zangari et al., 2023). If a fiber is too long to be completely ingested, the macrophage only surrounds a part of it, giving rise to a micro-environment in the space between the macrophage and the fiber. The macrophage seals off this space, forming a separate compartment from which the exocytosed lysosomal content and ferritin cannot escape, and in which the AB begin to form (Koerten et al., 1990a). Conversely, when the fiber is short enough, it can be ingested by a single macrophage or giant cell. In this case, the AB forms inside a phagosome, which later matures to a phagolysosome. In both cases the ferritin’s mineral core, ferrihydrite, is exposed to pH falling in the range 4.5–5.5 (Innes et al., 2021), corresponding to the pH range of maximum goethite formation. On the other hand, the physiological pH (~ 7.4) of the extracellular environment would instead favor the formation of hematite. However, the alveolar macrophages exposed to asbestos are characterized by a high turnover due to ferroptotic cell death (Ito et al., 2021). This leads to successive attempts of fiber internalization by new macrophages, which would repeatedly lead to a drop in pH to the acidic conditions in the microenvironment created around the fiber, favoring the formation of goethite instead of hematite. Thousands of these cycles would result in the formation of goethite-bearing AB.

**Fig. 5:**
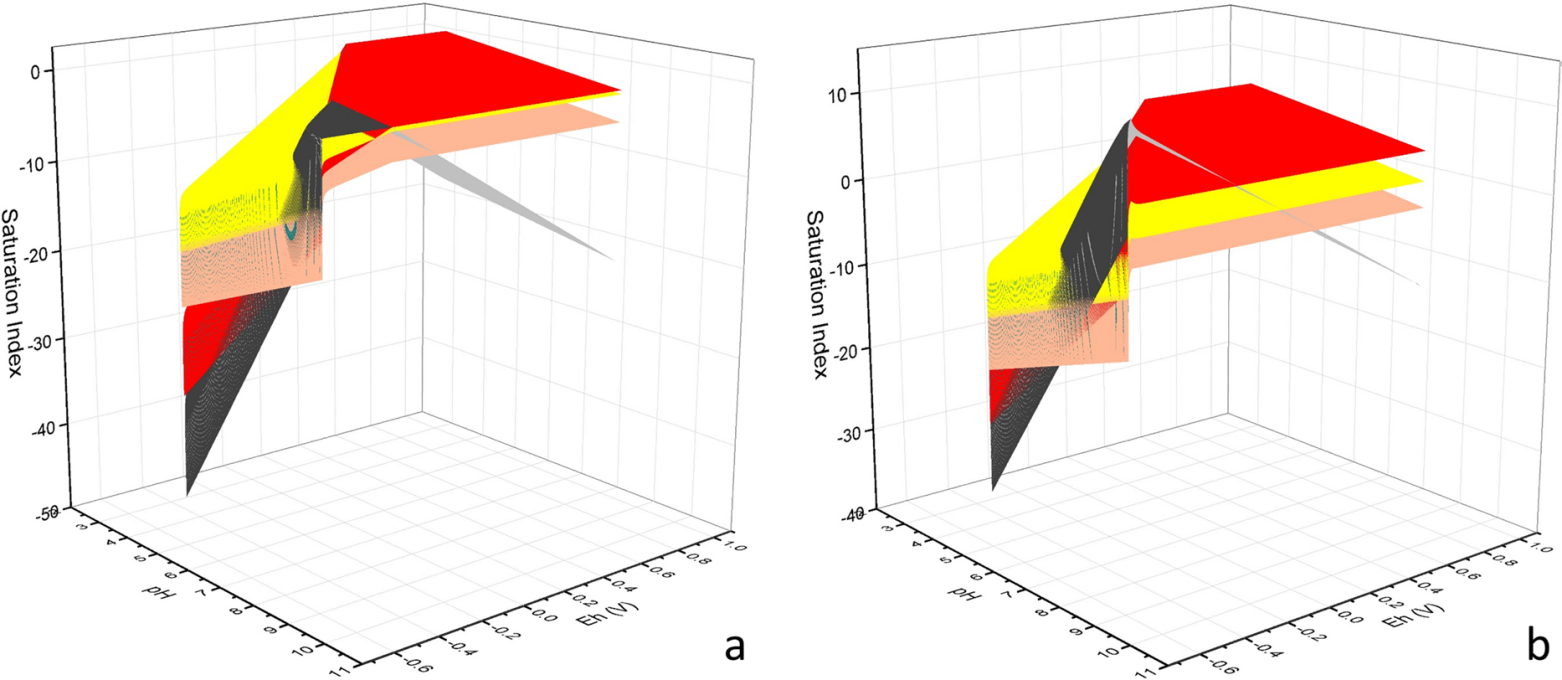
Saturation Index (SI) diagram *versus* pH and Eh. The different hypersurfaces refer to the different solid Fe species according to the coding: red—hematite, yellow—goethite, dark grey—magnetite, and ochre—ferrihydrite. When no constraints are imposed to the system, SI is negative for all phases except hematite in the entire pH—Eh range (**a**), meaning that no other stable Fe precipitates are thermodynamically allowed. On the contrary, when precipitation is allowed only for ferrihydrite (**b**), the residual amount of Fe in equilibrium with the ferrihydrite solid precipitate is supersaturated with respect to goethite, magnetite, hematite, which can thus start to precipitate

Thermodynamic geochemical calculations were performed to study the stability of the relevant Fe-oxides and oxyhydroxides (magnetite, hematite, goethite, and 2- and 6-lines ferrihydrite) starting from a concentration of Fe^2+^ in solution typical of human blood. The results are summarized in Fig. 5, where the saturation index (SI), which is proportional to the species stability, is plotted against the pH value and the chemical potential (Eh). In particular, when no constraints were imposed on the system (Fig. 5a), the results point out the absence of stable Fe^2+^ precipitates, and indicate the following order in terms of decreasing stability (i.e., SI): hematite > magnetite > goethite > ferrihydrite (at high pH and low Eh values magnetite is more stable than hematite, whereas, at high pH and high Eh, hematite is less stable than magnetite). This trend reflects in the Pourbaix prevalence diagram (Figure S9), where only hematite and magnetite are present. On the other hand, goethite and ferrihydrite can be present only if the precipitation of hematite and magnetite is inhibited, as shown in the Pourbaix diagrams for goethite and ferrihydrite (Figures S10 and S11).

An attempt was made to force the system in a situation closer to the actual system, where ferrihydrite is already present in the batch of reactants because of ferritin. For this, the precipitation was allowed only for ferrihydrite. The resulting calculations show that the residual amount of Fe in equilibrium with the ferrihydrite solid precipitate is still supersaturated with respect to goethite, hematite, and magnetite (Fig. 5b). The results of thermodynamic calculations indicate that if the starting Fe species is ferrihydrite, the system should evolve into more stable and well-crystalline Fe species, such as goethite, hematite, and magnetite. The fact that in this work only ferrihydrite and goethite were observed, suggests that a relevant kinetic control of the precipitation and transformation of the solid phases occurs. In fact, long term batch experiments performed by Schwertmann on pure synthetic ferrihydrite showed that its rate of transformation to hematite is slow (Schwertmann et al., 2004), with around 7% of the initial ferrihydrite remaining after almost four years at pH 4 and 25 °C. Considering that the AB studied in this work have aged well longer than four years, a complete transformation to goethite or hematite should eventually have occurred. XAS data, instead, showed that about 70% of the total Fe content is still in the form of ferrihydrite. However, in another study (Carlson & Schwertmann, 1981) on natural ferrihydrite samples from Fe-bearing spring waters in Finland, it was observed that the transformation of ferrihydrite into more stable forms was highly reduced with respect to the experiments performed with pure synthetic ferrihydrite solutions. The authors concluded that the presence of dissolved compounds with high affinity for ferrihydrite, such as silicates and phosphates, might inhibit the transformation of ferrihydrite to goethite or hematite, as evidenced by the detection of Si–O–Fe bonds in the system.

Accordingly, the transformation of ferrihydrite may have been slowed down by its adsorption on the surface of asbestos, which are silicate minerals. Moreover, high amounts of P, Ca, and Mg in the fibers coating were observed by XRF mapping (Bardelli et al., 2017; Pascolo et al., 2011), which may also interfere with the transformation of ferrihydrite predicted by the thermodynamics.

The discovery of the presence of a significant fraction of goethite in the AB has toxicological implications. In fact, goethite has been shown to be the most cytotoxic among Fe-oxides, being able to induce a higher generation of ROS with respect to hematite, maghemite, and magnetite (Huang et al., 2019). Goethite nanoparticles have also been shown to be cytotoxic and genotoxic in a recent study comparing in vitro the effect of goethite, glyphosate, and Roundup® on liver cell lines (Da Silva et al., 2020). Ferrihydrite, on the other hand, has been shown to be more biocompatible, causing no significant toxic side effects (Chilom et al., 2017; Zhang et al., 2021). The cytotoxic properties of the AB may (also) be attributed to the presence of the highly reactive goethite species, although further research is needed to support this assumption.

### Supplementary Information

Below is the link to the electronic supplementary material.

Supplementary file1 (PDF 4373 kb)

## References

[CR1] Bardelli F, Brun F, Capella S, Bellis D, Cippitelli C, Cedola A, Belluso E (2021). Asbestos bodies count and morphometry in bulk lung tissue samples by non-invasive X-ray micro-tomography. Scientific Reports.

[CR2] Bardelli F, Brun F, De Panfilis S, Cloetens P, Capella S, Belluso E, Bellis D, Di Napoli A, Cedola A (2021). Chemo-physical properties of asbestos bodies in human lung tissues studied at the nano-scale by non-invasive, label free X-ray imaging and spectroscopic techniques. Toxicology Letters.

[CR3] Bardelli F, Veronesi G, Capella S, Bellis D, Charlet L, Cedola A, Belluso E (2017). New insights on the biomineralisation process developing in human lungs around inhaled asbestos fibres. Scientific Reports.

[CR4] Blanc P, Lassin A, Piantone P, Azaroual M, Jacquemet N, Fabbri A, Gaucher EC (2012). Thermoddem: A geochemical database focused on low temperature water/rock interactions and waste materials. Applied Geochemistry.

[CR5] Bloise A, Punturo R, Catalano M, Miriello D, Cirrincione R (2016). Naturally occurring asbestos (NOA) in rock and soil and relation with human activities: The monitoring example of selected sites in Calabria (southern Italy). Italian Journal of Geosciences.

[CR6] Borelli V, Brochetta C, Melato M, Rizzardi C, Polentarutti M, Busatto C, Vita F, Abbate R, Gotter R, Zabucchi G (2007). A procedure for the isolation of asbestos bodies from lung tissue by exploiting their magnetic properties: A new approach to asbestos body study. Journal of Toxicology and Environmental Health. Part A.

[CR7] Bursi Gandolfi N, Gualtieri AF, Pollastri S, Tibaldi E, Belpoggi F (2016). Assessment of asbestos body formation by high resolution FEG-SEM after exposure of Sprague-Dawley rats to chrysotile, crocidolite, or erionite. Journal of Hazardous Materials.

[CR8] Carlson L, Schwertmann U (1981). Natural ferrihydrites in surface deposits from Finland and their association with silica. Geochimica Et Cosmochimica Acta.

[CR9] Chasteen ND, Harrison PM (1999). Mineralization in ferritin: An efficient means of iron storage. Journal of Structural Biology.

[CR10] Chilom CG, Găzdaru DM, Bălăsoiu M, Bacalum M, Stolyar SV, Popescu AI (2017). Biomedical application of biogenic ferrihydrite nanoparticles. Romanian Journal of Physics.

[CR11] Craighead JE, Abraham JL, Churg A, Green FH, Kleinerman J, Pratt PC, Seemayer TA, Vallyathan V, Weill H (1982). The pathology of asbestos-associated diseases of the lungs and pleural cavities: Diagnostic criteria and proposed grading schema. Report of the Pneumoconiosis Committee of the College of American Pathologists and the National Institute for Occupational Sa. Archives of Pathology & Laboratory Medicine.

[CR12] Croce A, Musa M, Allegrina M, Trivero P, Rinaudo C (2013). Environmental scanning electron microscopy technique to identify asbestos phases inside ferruginous bodies. Microscopy and Microanalysis.

[CR13] Cudennec Y, Lecerf A (2006). The transformation of ferrihydrite into goethite or hematite, revisited. Journal of Solid State Chemistry.

[CR14] Da Silva NDG, Carneiro CEA, Campos EVR, de Oliveira JL, Risso WE, Fraceto LF, Zaia DAM, Martinez CBR (2020). Interference of goethite in the effects of glyphosate and Roundup® on ZFL cell line. Toxicology in Vitro.

[CR15] Das S, Hendry MJ, Essilfie-Dughan J (2011). Transformation of two-line ferrihydrite to goethite and hematite as a function of pH and temperature. Environmental Science and Technology.

[CR16] Davis JM (1964). The ultrastructure of asbestos bodies from human lung. British Journal of Experimental Pathology.

[CR17] Di Giuseppe D, Zoboli A, Vigliaturo R, Gieré R, Bonasoni MP, Sala O, Gualtieri AF (2019). Mineral fibres and asbestos bodies in human lung tissue: A case study. Minerals.

[CR18] Fubini B, Mollo L (1995). Role of iron in the reactivity of mineral fibers. Toxicology Letters.

[CR19] Ghio AJ, Stonehuerner J, Richards J, Devlin RB (2008). Iron homeostasis in the lung following asbestos exposure. Antioxidants & Redox Signaling.

[CR20] Giacobbe C, Di Giuseppe D, Zoboli A, Lassinantti Gualtieri M, Bonasoni P, Moliterni A, Corriero N, Altomare A, Wright J, Gualtieri AF (2021). Crystal structure determination of a lifelong biopersistent asbestos fibre using single-crystal synchrotron X-ray micro-diffraction. IUCrJ.

[CR21] Gloyne SR (1931). The formation of the asbestosis body in the lung. Tubercle.

[CR22] Governa M, Amati M, Fontana S, Visona I, Botta GC, Mollo F, Bellis D, Bo P (1999). Role of iron in Asbestos-Body-induced oxidant radical generation. Journal of Toxicology and Environmental Health, Part A.

[CR23] Gualtieri AF, Bursi Gandolfi N, Pollastri S, Burghammer M, Tibaldi E, Belpoggi F, Pollok K, Langenhorst F, Vigliaturo R, Dražić G (2017). New insights into the toxicity of mineral fibres: A combined in situ synchrotron μ-XRD and HR-TEM study of chrysotile, crocidolite, and erionite fibres found in the tissues of Sprague-Dawley rats. Toxicology Letters.

[CR24] Hamilton RF, Li L, Iyer R, Holian A (1996). Asbestos induces apoptosis in human alveolar macrophages. American Journal of Physiology.

[CR25] Harrison P, Arosio P (1996). The ferritins: Molecular properties, iron storage function and cellular regulation. Biochimica Et Biophysica Acta.

[CR26] Harrison PM, Fischbach FA, Hoy TG, Haggis GH (1967). Ferric oxyhydroxide core of ferritin. Nature.

[CR27] Huang HH (2016). The Eh-pH diagram and its advances. Metals.

[CR28] Huang M, Xiang W, Zhou T, Mao J, Wu X, Guo X (2019). The critical role of the surface iron-oxalate complexing species in determining photochemical degradation of norfloxacin using different iron oxides. Science of the Total Environment.

[CR29] Innes E, Yiu HHP, McLean P, Brown W, Boyles M (2021). Simulated biological fluids—A systematic review of their biological relevance and use in relation to inhalation toxicology of particles and fibres. Critical Reviews in Toxicology.

[CR30] Isaure M-P, Laboudigue A, Manceau A, Sarret G, Tiffreau C, Trocellier P, Lamble G, Hazemann J-L, Chateigner D (2002). Quantitative Zn speciation in a contaminated dredged sediment by μ-PIXE, μ-SXRF, EXAFS spectroscopy and principal component analysis. Geochimica Et Cosmochimica Acta.

[CR31] Ito F, Kato K, Yanatori I, Murohara T, Toyokuni S (2021). Ferroptosis-dependent extracellular vesicles from macrophage contribute to asbestos-induced mesothelial carcinogenesis through loading ferritin. Redox Biology.

[CR32] Jia Y, Xu L, Wang X, Demopoulos GP (2007). Infrared spectroscopic and X-ray diffraction characterization of the nature of adsorbed arsenate on ferrihydrite. Geochimica Et Cosmochimica Acta.

[CR33] Koerten, H. K., Hazekamp, J., Kroon, M., & Daems, W. T., (1990b). Asbestos body formation and iron accumulation in mouse peritoneal granulomas after the Introduction of Crocidolite Asbestos Fibers *136*, 141–157. PMC1877471 2153345

[CR34] Koerten HK, de Bruijn JD, Daems WT (1990). The formation of asbestos bodies by mouse peritoneal macrophages: An in vitro study. The American Journal of Pathology.

[CR35] Lee PA, Citrin PH, Eisenberger P, Kincaid BM (1981). Extendend X-ray absorption fine structure-its strenghts and limitations as a structural tool. Review of Modern Physics.

[CR36] Lund LG, Williams MG, Dodson RF, Aust AE (1994). Iron associated with asbestos bodies is responsible for the formation of single strand breaks in phi X174 RFI DNA. Occupational and Environmental Medicine.

[CR37] McLemore TL, Mace ML, Roggli V, Marshall MV, Lawrence EC, Wilson RK, Martin RR, Brinkley BR, Greenberg SD (1980). Asbestos body phagocytosis by human free alveolar macrophages. Cancer Letters.

[CR38] Nakamura E, Makishima A, Hagino K, Okabe K (2009). Accumulation of radium in ferruginous protein bodies formed in lung tissue: Association of resulting radiation hotspots with malignant mesothelioma and other malignancies. Proceedings of the Japan Academy, Series B.

[CR39] Overall Evaluations of Carcinogenicity. (1987). *IARC monographs on the evaluation of the carcinogenic risks to humans—overall evaluations of carcinogenicity: An Updating of IARC Mongraphs*. International Agency for Research on Cancer.

[CR40] Parkhurst DL, Appelo CAJ (1999). User’s guide to PHREEQC (Version 2): A computer program for speciation, batch-reaction, one-dimensional transport, and inverse geochemical calculations. Water-Resources Investigations Report.

[CR41] Pascolo L, Gianoncelli A, Kaulich B, Rizzardi C, Schneider M, Bottin C, Polentarutti M, Kiskinova M, Longoni A, Melato M (2011). Synchrotron soft X-ray imaging and fluorescence microscopy reveal novel features of asbestos body morphology and composition in human lung tissues. Particle and Fibre Toxicology.

[CR42] Pascolo L, Gianoncelli A, Schneider G, Salomé M, Schneider M, Calligaro C, Kiskinova M, Melato M, Rizzardi C (2013). The interaction of asbestos and iron in lung tissue revealed by synchrotron-based scanning X-ray microscopy. Scientific Reports.

[CR43] Peto J, Decarli A, La Vecchia C, Levi F, Negri E (1999). The European mesothelioma epidemic. British Journal of Cancer.

[CR44] Pollastri S, Gualtieri AF, Vigliaturo R, Ignatyev K, Strafella E, Pugnaloni A, Croce A (2016). Stability of mineral fi bres in contact with human cell cultures. An in situ m XANES, m XRD and XRF iron mapping study. Chemosphere.

[CR45] Pooley FD (1972). Asbestos bodies, their formation, composition and character. Environmental Research.

[CR46] Ravel B, Newville M (2005). ATHENA, ARTEMIS, HEPHAESTUS: Data analysis for X-ray absorption spectroscopy using IFEFFIT. Journal of Synchrotron Radiation.

[CR47] Rinaudo C, Croce A, Musa M, Fornero E, Allegrina M, Trivero P, Bellis D, Sferch D, Toffalorio F, Veronesi G, Pelosi G (2010). Study of inorganic particles, fibers, and asbestos bodies by variable pressure scanning electron microscopy with annexed energy dispersive spectroscopy and micro-Raman spectroscopy in thin sections of lung and pleural plaque. Applied Spectroscopy.

[CR48] Schwertmann U, Stanjek H, Becher H-H (2004). Long-term in vitro transformation of 2-line ferrihydrite to goethite/hematite at 4, 10, 15 and 25°C. Clay Minerals.

[CR49] Shavelle R, Vavra-Musser K, Lee J, Brooks J (2017). Life expectancy in pleural and peritoneal mesothelioma. Lung Cancer International.

[CR50] Suzuki Y, Churg J (1969). Structure and development of the asbestos body. The American Journal of Pathology.

[CR51] Wagner JC, Berry G, Skidmore JW, Timbrell V (1974). The effects of the inhalation of asbestos in rats. British Journal of Cancer.

[CR52] Zangari M, Borelli V, Bernareggi A, Zabucchi G (2023). Asbestos fibers promote iron oxidation and compete with apoferritin enzymatic activity. Journal of Toxicology and Environmental Health: Part a: Current Issues.

[CR53] Zhang R, Chen L, Liang Q, Xi J, Zhao H, Jin Y, Gao X, Yan X, Gao L, Fan K (2021). Unveiling the active sites on ferrihydrite with apparent catalase-like activity for potentiating radiotherapy. Nano Today.

