# Supplementary material for: Closing the knowledge gap on the composition of the asbestos bodies
Source: Environ Geochem Health. 2023 Apr 14;45(7):5039–51. doi: 10.1007/s10653-023-01557-0 (PMC10310571; doi:10.1007/s10653-023-01557-0)
Supplement: Supplementary file 1 — Supplementary file1 (PDF 4373 kb) [file 10653_2023_1557_MOESM1_ESM.pdf]

Closing the knowledge gap on the composition of the asbestos bodies

Bardelli F.[1,2*], Giacobbe C.[3,4], Ballirano P.[5], Borelli V.[6], Di Benedetto F.[7], Montegrossi G.[8], D. Bellis[2], Pacella A.[5]

[1] National Research Council, Institute of Nanotechnology (CNR-Nanotec), Roma, Italy

[2] Centre for the Study of Asbestos and other Toxic Particulate, University of Torino, Torino, Italy

[3] Xenocs SAS, Grenoble, France

[4] European Synchrotron Radiation Facility, Grenoble, France

[5] La Sapienza University, Department of Earth Sciences, Roma, Italy

[6] University of Trieste, Department of Physiology and Pathology, Trieste, Italy

[7] University of Ferrara, Department of Earth Sciences, Ferrara, Italy

[8] National Research Council, Institute of Geoscience and Earth Resources (CNR-IGG), Firenze, Italy

[*] fabrizio.bardelli@cnr.it

Supporting Information

Tables S1 and S2

Figures S1 – S11

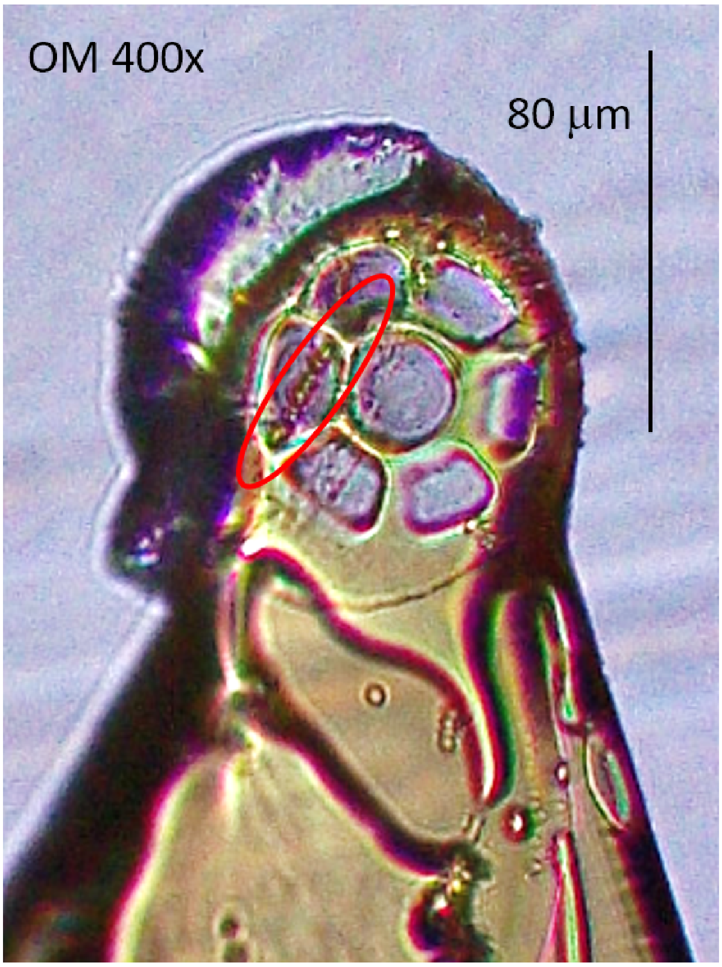

Figure S1. Optical microscopy image of the Kapton® sample holder (micromesh 80/15 from MiTeGen) used for XRD measurements with the tissue fragment cut with a laser microdissector and then glued on the sample holder. The red ellipsoid highlights the AB in the lung tissue fragment.

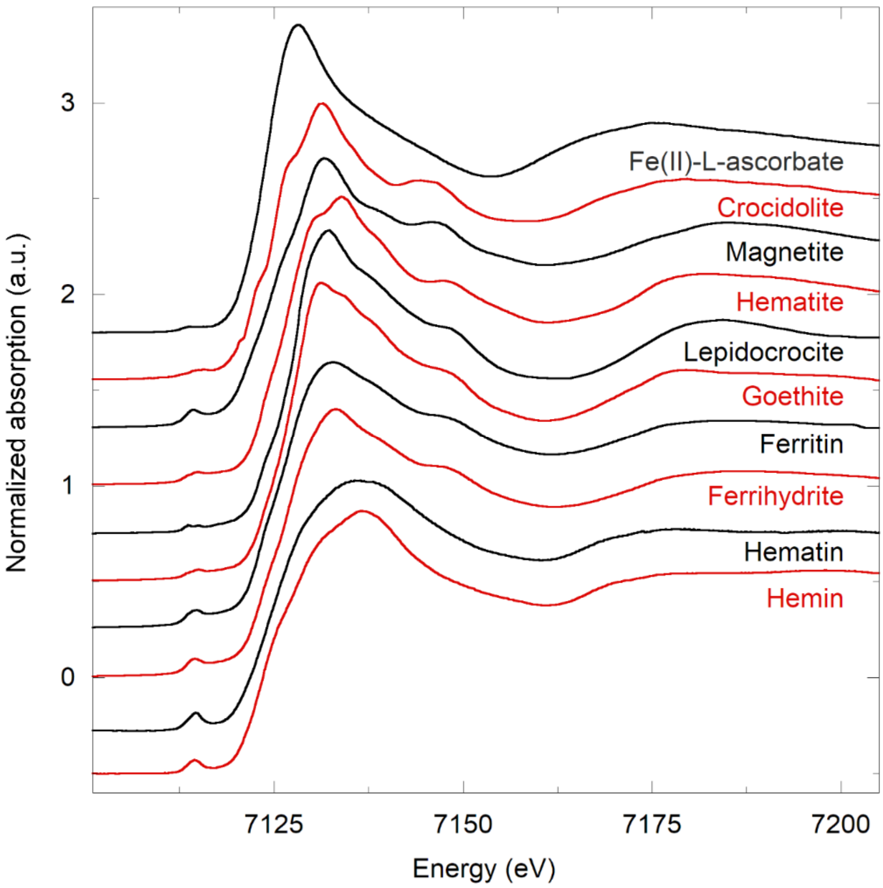

Figure S2. XANES spectra of all Fe-references. The ferrihydrite, goethite, ferritin, hematite, crocidolite, lepidocrocite, and magnetite spectra were acquired at the B18 beamline at the Diamond light source. The other references (hematin, hemin, and Fe(II)-L-ascorbate) were acquired at the ID21 at the ESRF, along with the spectra of the AB. The same monochromator crystals (Si 111) and energy steps (~0.3 eV) were used for all references, ensuring a comparable energy resolution.

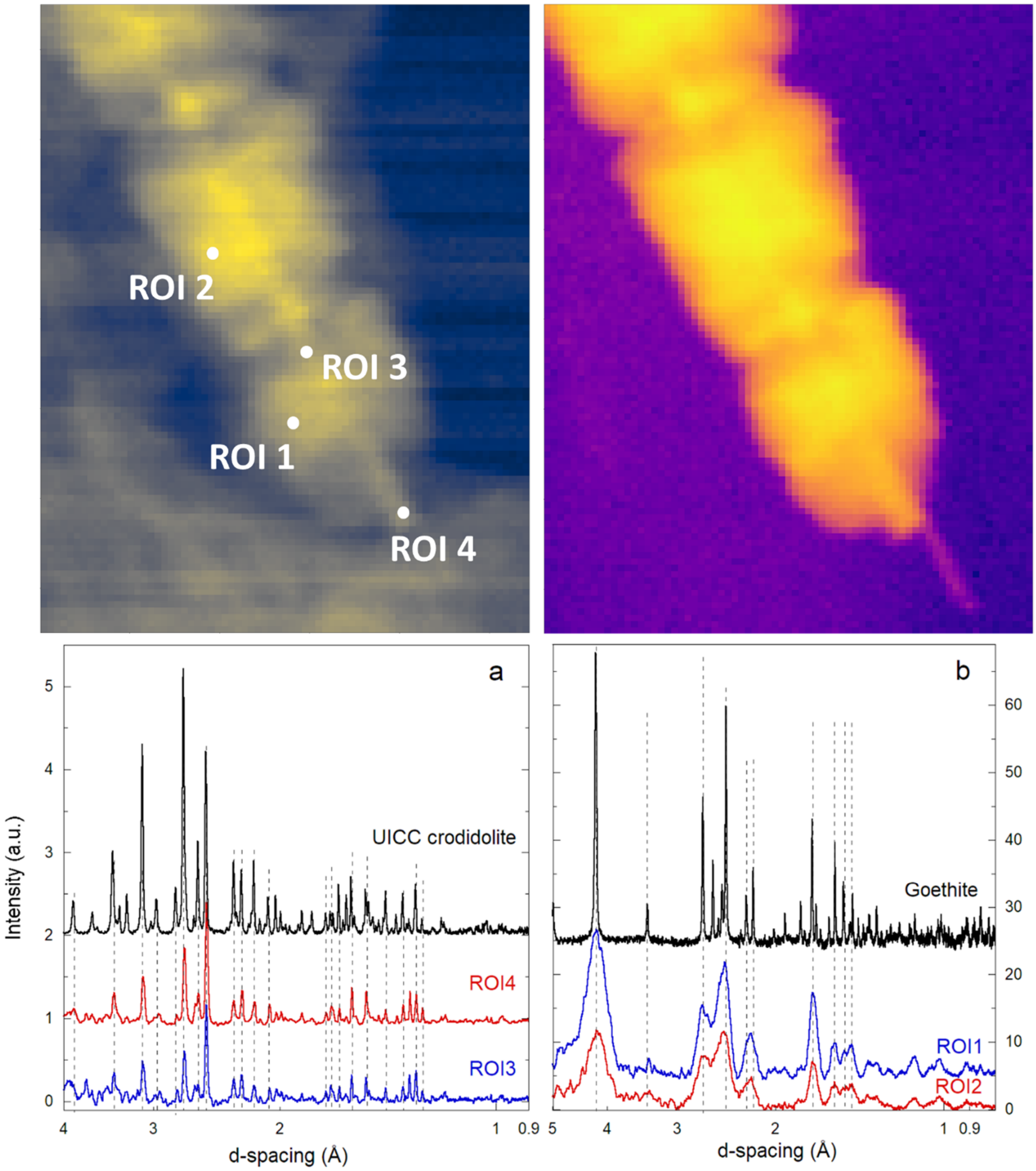

Figure S3. Left upper panel: map of the total XRD signal of a representative AB embedded in the lung tissue. A diffraction image is associated with each pixel. The labelled white points correspond to point XRD acquisitions. Right upper panel: XRF map acquired at simultaneously to the XRD map, showing the distribution of Fe in the AB. The incident energy was 43.457 keV and the X-ray beam spot size was 0.15 μm x 0.15 μm. Lower panels: a) XRD patterns acquired on uncoated parts of the AB (ROI3 and ROI4) and of the UICC crocidolite standard. b) XRD patterns acquired at ROI1 and ROI2 and of the goethite reference. The dashed vertical lines highlight the main peak correspondences between data and references. The patterns of crocidolite and goethite were acquired in transmission mode on a capillary at a wavelength of 1.5406 Å. The patterns have been background subtracted (using the pattern of an empty sample holder and a polynomial function to fit the residual background), rescaled, and vertically shifted for easy of view and comparison. The x-axes (d-spacing) are in logarithmic scale.

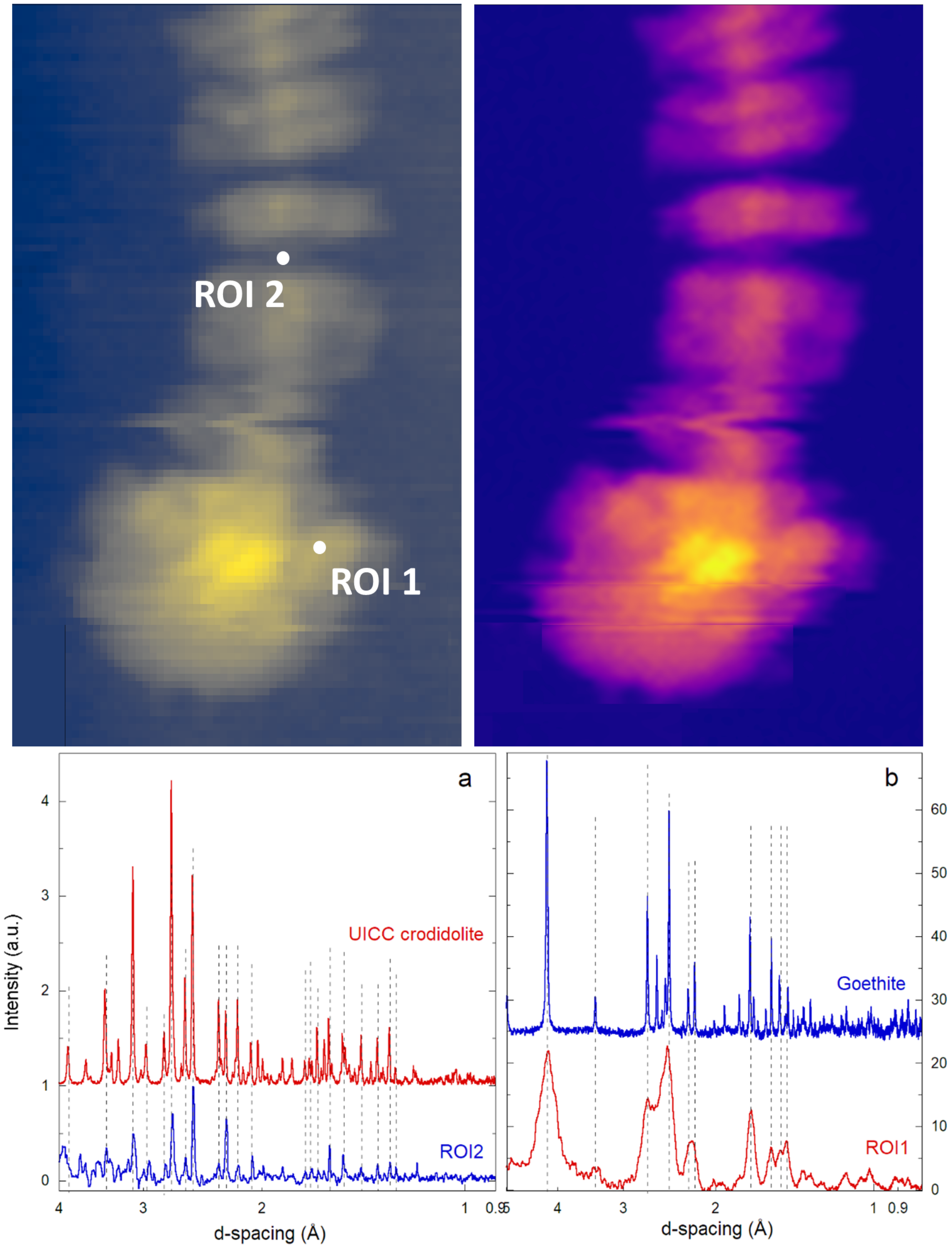

Figure S4. Left upper panel: map of the total XRD signal of a representative AB embedded in the lung tissue. A diffraction image is associated with each pixel. The labelled white points correspond to point XRD acquisitions. Right upper panel: XRF map acquired at simultaneously to the XRD map, showing the distribution of Fe in the AB. The incident energy was 43.457 keV and the X-ray beam spot size was 0.15 μm x 0.15 μm. Lower panels: a) XRD patterns acquired on an uncoated part of the AB (ROI2) and of the UICC crocidolite standard. b) XRD patterns of ROI1 and of the goethite reference. The patterns of crocidolite and goethite were acquired in transmission mode on a capillary at a wavelength of 1.5406 Å. The dashed vertical lines highlight the main peak correspondences between data and references. The patterns have been background subtracted

(using the pattern of an empty sample holder and a polynomial function to fit the residual background), rescaled, and vertically shifted for easy of view and comparison. The x-axes (d-spacing) are in logarithmic scale.

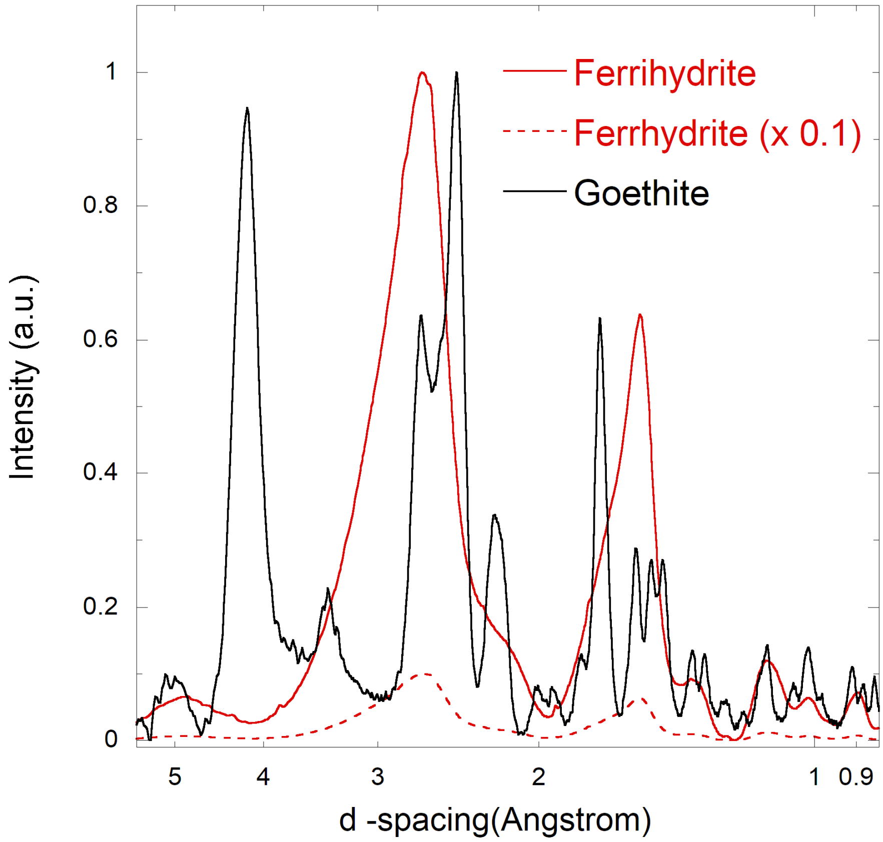

Figure S5. Representative XRD pattern of an AB and of the ferrihydrite reference normalized to the maximum intensities. As can be seen, the synthetic ferrihydrite used in this study is closer to a 6-line than 2-line structure. The fact that ferrihydrite was not observed in the XRD patterns, but was revealed by XAS data, can be explained by the actual relative intensities of the samples and ferrihydrite patterns, which could be similar to the ones highlighted by the dashed line (ferrihydrite pattern with intensities divided by 10). It must be noted that the crystallinity of the actual ferrihydrite present in the AB is probably much lower (2-lines) than that of the ferrihydrite reference (6-lines), with broader and less intense XRD peaks. The x-axis is in logarithmic scale.

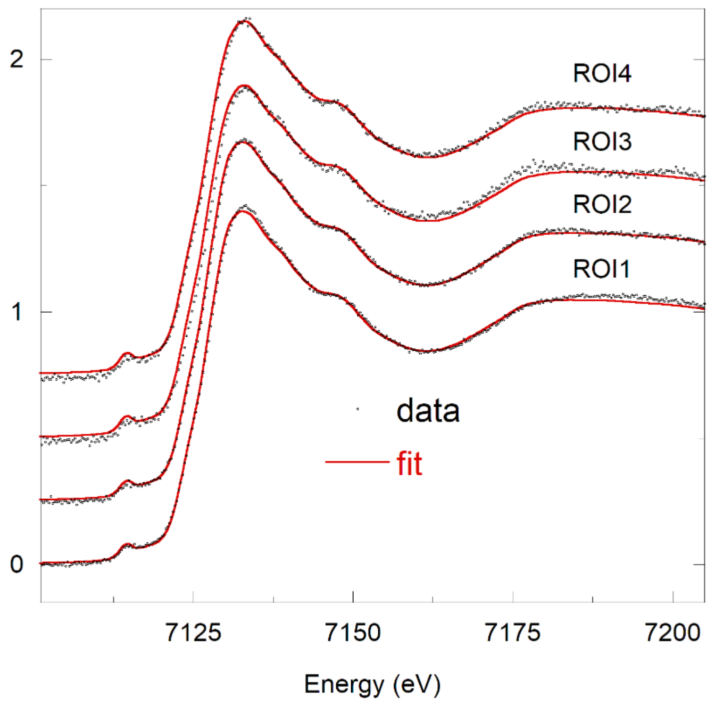

Figure S5. Best LCF of the four ROI of a representative AB performed using the ferrihydrite and goethite references. The LCF shown in Figure SX were performed on the spectrum resulting from the average of these four ROI spectra. The numerical results are reported in **Table S1**.

Table S1. Ferrihydrite and goethite fractions of the four ROI of a representative AB calculated from LCF. The sum of the fraction and the χ_ν^2 and R statistical indicators are also reported. The best LCF for the spectrum resulting from the average of the four ROI spectra is reported in the last line.

	Ferrihydrite %	Goethite %	Σ	R	χ_ν^2
ROI1	63	34	97	0.00052	0.0356
ROI2	60	38	98	0.00060	0.0426
ROI3	74	24	98	0.00309	0.2158
ROI4	75	23	97	0.00140	0.0985
Average ± SD	68 ± 8	30 ± 7			
ROI1+ROI2+ROI3+ROI4	68	31	99	0.00083	0.0339

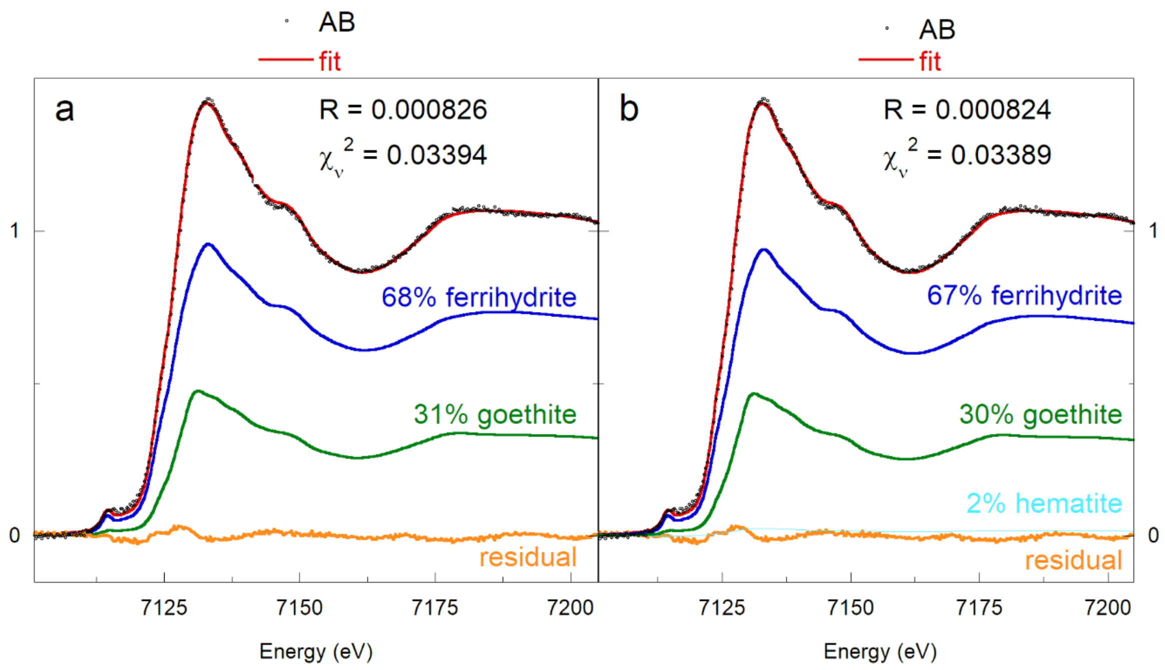

Figure S7. Comparison between: (a) the best 2-component LCF (ferrihydrite and goethite), and (b) the best 3-component LCF (ferrihydrite, goethite, and hematite). As it can be seen from the figure, the match of both fits with the spectrum of the AB is nearly identical by eye. The contribution of the hematite fraction is at the level of the residual, and that the statistical indicators R and reduced χ^2 decrease only negligibly in the 3-component fit, indicating that the 2-component fit is more suitable to model the data.

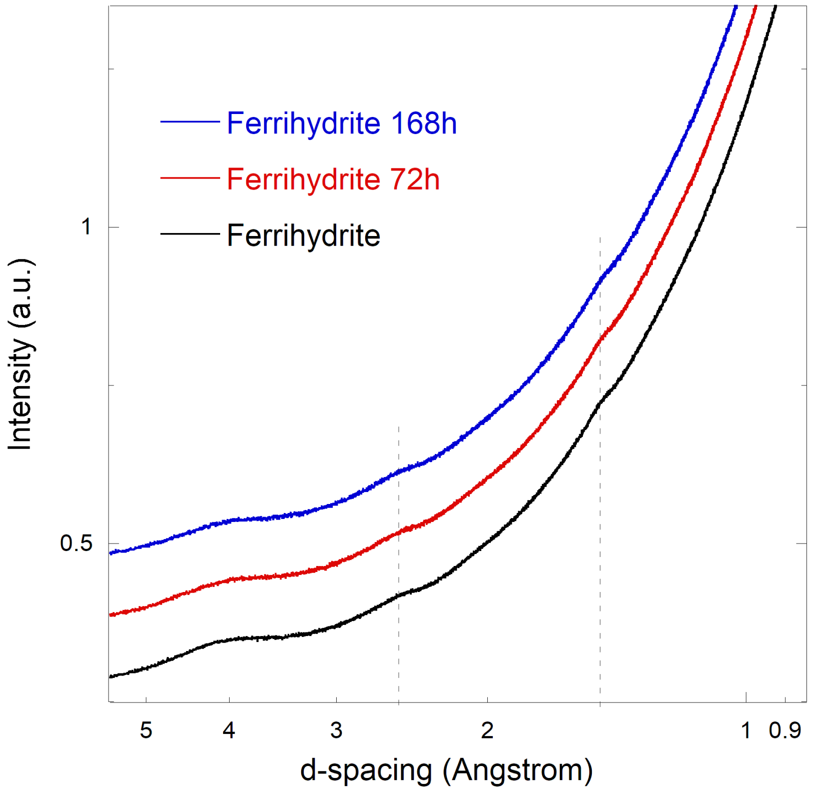

Figure S8. Comparison between the pristine synthetic ferrihydrite and those treated as described in the following, to simulate the sample preparation method (fixation of biological tissues and inclusion in paraffin). Ferrihydrite was treated using the following protocol:

- 72h or 168h in buffered formalin (10%)
- 1h in ethanol (70%)
- 45 minutes in ethanol (95%)
- 1.5h in ethanol (100%)
- 1h in xylene (100%)

The samples at 72h and 168h were then recovered by filtration and heated for 12h at 60°C before XRD measurements. As shown in the figure, the comparison with the pristine ferrihydrite sample showed no formation of secondary phases (goethite or hematite, in particular). The vertical dashed lines highlight the major features of ferrihydrite at ~1.5 and ~ 2.5 Å. The measurements were acquired in transmission mode on a capillary, with a laboratory diffractometer at the Cu Kα wavelength. The x-axis is in logarithmic scale.

Table S2. Number XRD and XAS samples measured and usable XAS spectra or XRD patterns.

Beamline (technique)	x-ray beam spot size	Number of AB mapped by XRF	ROI (XAS)	ROI (XRD)
ID21 (XRF + XANES)	0.5μm x 0.5μm	8	8	-
ID11 (XRF + XRD)	0.15μm x 0.15μm	3	-	9

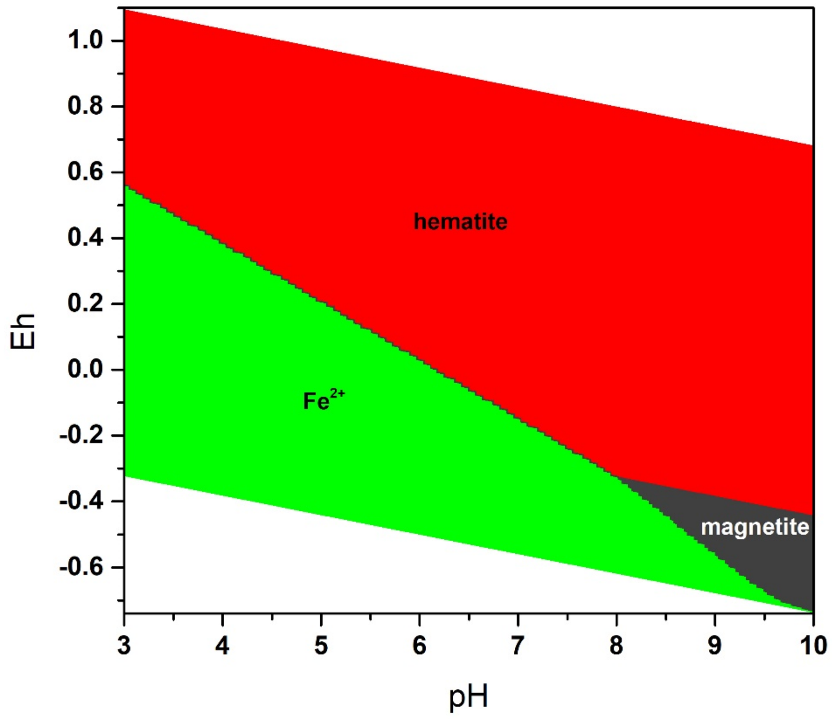

Figure S9. Pourbaix prevalence diagram of the Fe speciation under the chosen boundary conditions (see text for further information). The green regions indicate the stability field of Fe^{2+} and Fe^{3+} species in solution.

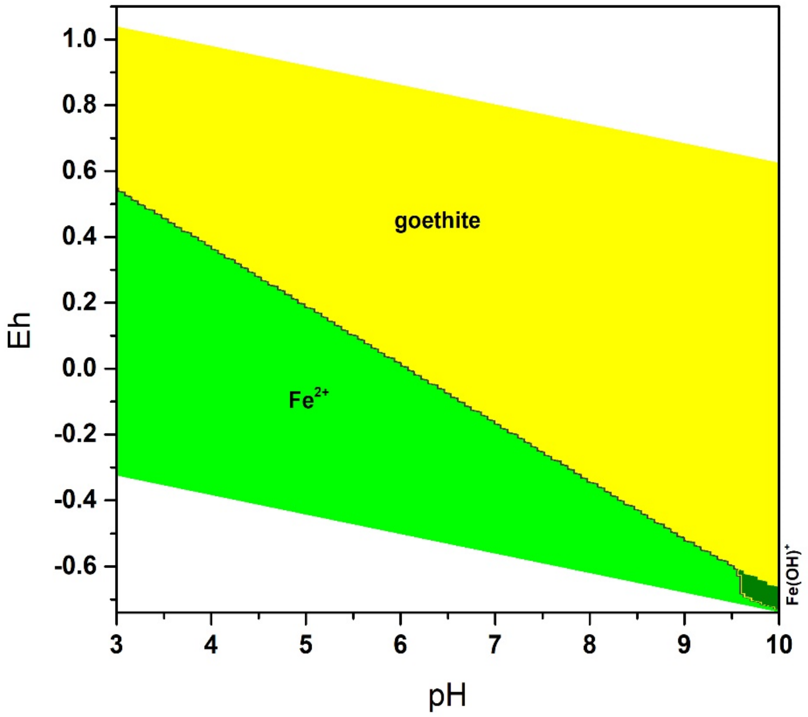

Figure S10. Pourbaix prevalence diagram of the Fe speciation obtained by preventing hematite and magnetite from precipitation, and calculated under the chosen boundary conditions (see text for further information). The green regions indicate the stability field of Fe^{2+} and Fe^{3+} species in solution.

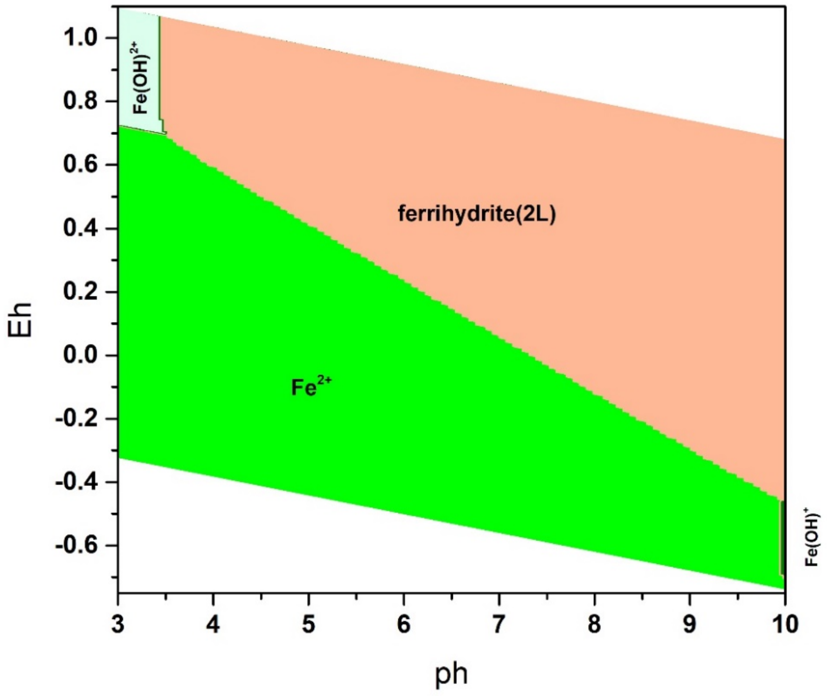

Figure S11. Pourbaix prevalence diagram of the Fe speciation obtained by preventing hematite, magnetite, and goethite from precipitation, and calculated under the chosen boundary conditions (see text for further information). The green regions indicate the stability field of Fe^{2+} and Fe^{3+} species in solution.
